# Natural killer cell-derived extracellular vesicles in cancer: mediators of intercellular communication and tumor immune signaling

**DOI:** 10.3389/fimmu.2026.1919867

**Published:** 2026-10-12

**Authors:** Jun Liu, Yancong Yang, Wenjun Zhang, Jianwu Liu, Yanyan Yu, Ye Song, Xiaofei Liao, Qiuhua Jiang, Hua Guo, Qianliang Huang

**Affiliations:** 1 Department of Neurosurgery, The 2nd Affiliated Hospital, Jiangxi Medical College, Nanchang University, Nanchang, Jiangxi, China; 2 Department of Neurosurgery, The Affiliated Ganzhou Hospital, Jiangxi Medical College, Nanchang University, Ganzhou, Jiangxi, China; 3 Department of Rehabilitation Medicine, The Affiliated Ganzhou Hospital, Jiangxi Medical College, Nanchang University, Ganzhou, Jiangxi, China; 4 Department of Neurosurgery, Guangzhou Women and Children’s Medical Center, Guangzhou, Guangdong, China; 5 Department of Pharmacy, Ganzhou People’s Hospital, Ganzhou, Jiangxi, China

**Keywords:** intercellular communication, miRNA-mediated regulation, natural killer cell-derived extracellular vesicles (NK-EVs), tumor immune signaling, tumor microenvironment

## Abstract

Natural killer (NK) cells are essential components of innate immunity and play a pivotal role in tumor surveillance through direct cytotoxicity and immune regulation. In recent years, NK-cell-derived extracellular vesicles (NK-EVs) have emerged as a promising cell-free therapeutic modality that preserves key antitumor functions of parental NK cells while overcoming several limitations associated with cell-based immunotherapy. As biologically derived nanovesicles, NK-EVs combine nanoscale delivery properties with a cell-free formulation format and generally low immunogenicity. However, although selected preclinical studies suggest tumor-interacting or target-cell-recognition properties, a broadly applicable intrinsic tumor-targeting capacity—and, in particular, intrinsic blood–brain barrier (BBB) penetration—has not been established for native NK-EVs. This review systematically summarizes the biological characteristics, biogenesis pathways, and molecular cargo of NK-EVs, with particular emphasis on their antitumor mechanisms. Current evidence supports perforin/granzyme-mediated cytotoxicity and Fas/Fas ligand (FasL) signaling as the most established mechanisms of NK-EVs-mediated tumor killing, whereas miRNA-mediated regulation and tumor microenvironment remodeling represent emerging functional pathways requiring further validation. By contrast, tumor necrosis factor-related apoptosis-inducing ligand (TRAIL)–death receptor 4/5 (DR4/DR5) signaling remains putative because direct NK-EVs-specific causal evidence is limited. Functionally investigated miRNAs and tumor-microenvironment effects represent context-dependent or emerging pathways whose generalizability across NK-cell sources and preparation conditions requires further validation. Importantly, recent advances in engineering strategies—including parental-cell priming, cargo loading, antigen-recognition engineering, tissue/barrier-targeting modification, biomimetic nanovesicle construction, and formulation-based stabilization. These approaches can alter cargo composition, recipient-cell recognition, or tissue-specific delivery through distinct mechanisms. Improvements achieved by engineered composite platforms should therefore not be conflated with intrinsic properties of native NK-EVs. Nevertheless, major translational challenges remain, including scalable manufacturing, purification and characterization standardization, mechanism-linked potency testing, biodistribution uncertainty, batch comparability, and product-specific regulatory considerations. Overall, NK-EVs should be viewed not only as potential cell-free therapeutic tools but also as specialized mediators of tumor–immune communication that integrate cytotoxic protein transfer, receptor-mediated signaling, RNA-based regulation, and microenvironmental crosstalk.

## Graphical Abstract

**Figure:**
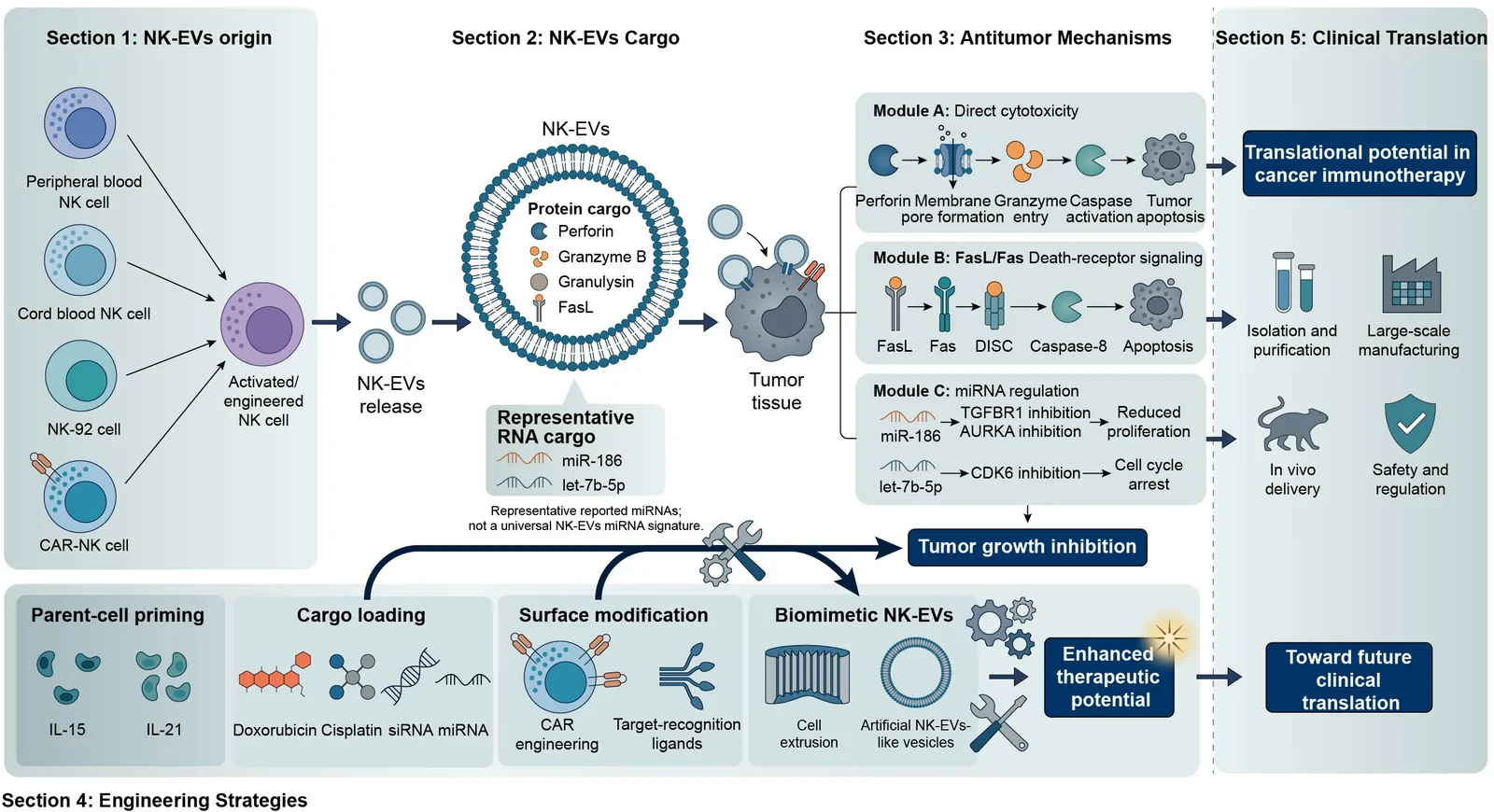
NK-EVs as mediators of tumor–immune communication and signaling in cancer. NK-EVs arise from biologically distinct NK-cell sources, while cytokine priming and CAR engineering represent functional or engineered states that can further modify EV composition. NK-EVs carry cytotoxic proteins, membrane-associated ligands, and context-dependent regulatory RNAs and can mediate tumor–immune communication through direct cytotoxicity, receptor signaling, post-transcriptional regulation, and selected recipient-cell effects. The graphical framework distinguishes stronger NK-EVs-specific evidence from preliminary or indirect mechanisms. Engineering approaches can alter antigen recognition, cargo, tissue/barrier delivery, manufacturability, or formulation stability through distinct mechanisms; properties conferred by engineered composite platforms should not be interpreted as intrinsic features of native NK-EVs. Translation remains limited by source and process heterogeneity, uncertain biodistribution, incomplete potency standardization, and product-specific regulatory requirements.

## 1 Introduction

In recent years, cancer immunotherapy has achieved significant progress in a subset of malignancies, yet solid tumors remain constrained by an immunosuppressive tumor microenvironment, limited response rates, and inefficient *in vivo* delivery of therapeutic molecules (1–3). How to enhance antitumor activity while simultaneously maintaining safety, stability, and effective *in vivo* accessibility remains a critical issue that requires continuous investigation in cancer therapy research (4–6). Natural killer (NK) cells are key effector cells of innate immunity that can recognize and eliminate abnormal cells without prior antigen sensitization and therefore have important roles in tumor surveillance and cancer immunotherapy (6–8). In addition to direct cytotoxicity mediated through cell–cell contact and cytokine secretion, recent studies have demonstrated that NK cells can also release extracellular vesicles (extracellular vesicles, EVs) (9–11). These NK-cell-derived extracellular vesicles (NK-EVs) can retain selected cytotoxic proteins, membrane-associated recognition molecules, and regulatory RNAs from their parental cells while offering a cell-free nanoscale format (10, 12, 13). Compared with adoptive NK-cell therapies, NK-EVs do not depend on long-term cellular persistence or sustained *in vivo* expansion; however, they also lack the continuous environmental sensing, proliferation, and dynamic adaptation of living NK cells (10, 12). In contrast to most EVs derived from non-immune cells, which primarily serve as delivery vehicles, NK-EVs are not merely passive carriers. Instead, they intrinsically carry and release immune effector molecules, such as perforin, granzymes, and certain membrane-associated functional proteins (14). It has been demonstrated that NK-EVs can exert direct or indirect inhibitory effects on tumor cells through the delivery of cytotoxic proteins, death receptor-related ligands, and various regulatory microRNAs (miRNAs) (10, 11, 15). At the same time, NK-EVs are also regarded as promising functional delivery platforms that can be utilized for drug loading, combination therapy, and targeted modification (12, 16, 17). However, from the perspective of overall research progress, the NK-EVs field is still largely confined to preclinical investigations. Key issues—including source heterogeneity, isolation and preparation methods, large-scale production, quality control, *in vivo* distribution, and long-term safety—have not yet reached a unified consensus. The immaturity of these fundamental aspects has significantly hindered their further clinical translation (3, 10, 12, 18). Based on these considerations, this review discusses NK-EVs as dynamic mediators of intercellular communication in cancer. We integrate current evidence on NK-EVs biogenesis, molecular cargo, cytotoxic effector signaling, death receptor pathways, miRNA-mediated post-transcriptional regulation, tumor microenvironment crosstalk, and engineering-based modulation of vesicle function. Rather than viewing NK-EVs only as cell-free immunotherapeutic tools, this review emphasizes their role as immune-cell-derived signaling vesicles that coordinate tumor–immune interactions. The overall conceptual framework of this review is summarized in the Graphical Abstract.

Several previous reviews have summarized the biological effects and therapeutic applications of NK-EVs, systematically evaluated preclinical safety and efficacy, or emphasized engineering and combination-treatment strategies (10, 12, 13, 16). The present review is differentiated by an evidence-weighted intercellular-communication framework that links NK-cell source and functional state to EV cargo, recipient-cell interactions, signaling consequences, engineering-dependent changes in recognition or biodistribution, and downstream manufacturing and regulatory requirements. Throughout the review, mechanistic claims are interpreted according to the level of NK-EVs-specific support: replicated functional evidence is distinguished from context-dependent, preliminary, indirect, or retracted evidence. This approach is intended to prevent mechanistic extrapolation from parental NK cells or unrelated EV systems and to identify which biological properties are intrinsic to NK-EVs versus conferred by engineered composite platforms.

## 2 Biological characteristics of NK-EVs

In the current literature, the use of terms such as “exosomes”, “small EVs”, and “extracellular vesicles” remains inconsistent, and in some studies, it is difficult to strictly distinguish different EV subpopulations in terms of isolation and characterization (19–21). According to the 2023 consensus issued by the International Society for Extracellular Vesicles (ISEV), in the absence of definitive evidence of biogenesis, it is not appropriate to define vesicles as exosomes solely based on particle size or isolation methods (22). Based on this consideration, the present study uniformly adopts the term “NK cell-derived extracellular vesicles (NK-EVs)” as a general designation, and interprets specific findings cautiously in light of their isolation and characterization evidence.

From a fundamental perspective, NK-EVs are membrane-bound nanostructures derived from immune cells, sharing the general characteristics of EVs. Specifically, they are enclosed by a lipid bilayer and can carry a diverse array of bioactive molecules, including proteins, lipids, and nucleic acids, thereby participating in intercellular communication (3, 23). Early studies once regarded EVs as byproducts generated during cellular metabolism; however, increasing evidence in recent years suggests that NK-EVs should be understood as functional outputs regulated by the state of the parental cells, rather than passively released residual structures (24–26). Their molecular composition is not static but varies in response to the activation status of NK cells, stimulation conditions, culture environment, and EV preparation workflow (24, 27, 28). Importantly, biological source and engineered or activation state should be distinguished. Primary sources used in NK-EVs research include peripheral-blood NK cells, cord-blood NK cells, and NK-cell lines such as NK-92, whereas cytokine priming and CAR engineering represent functional or engineered states rather than independent biological sources (7, 12, 29, 30) (**Figure 1**; **Table 1**). The major biological characteristics and translational features associated with these sources are summarized in **Table 1**. These different sources vary in expansion capacity, homogeneity, manufacturability, and potential safety. Therefore, the choice of source is not merely a “material difference,” but rather a. fundamental variable that determines functional stability and the feasibility of subsequent clinical translation (12, 31). In terms of biogenesis, NK-EVs generally include exosome-like vesicles formed through the endosomal pathway, as well as microvesicles generated through direct budding of the plasma membrane (23, 24, 32). The former is closely associated with multivesicular body formation and the endosomal sorting complex required for transport, whereas the latter is related to changes in membrane tension, Ca²^+^ signaling, and cytoskeletal remodeling (33, 34). However, in practical research, these subpopulations exhibit substantial overlap in size range, surface markers, and functional characteristics. Therefore, relying solely on biogenetic pathways is insufficient to accurately define their functional classification. With regard to molecular composition, NK-EVs can, to a certain extent, reflect the effector status of their parental cells. Cytotoxic molecules such as perforin, granzymes, and granulysin have been detected in NK-EVs, indicating their capacity to damage target cells through the perforin/granzyme axis (10, 11, 15, 35, 36). At the same time, the presence of membrane proteins such as FasL suggests that they may participate in death receptor-related signaling pathways (35, 37) (**Figure 1**). In addition to proteins, the miRNAs carried by NK-EVs also play important functional roles. These small nucleic acids can enter recipient cells and regulate gene expression, thereby influencing cell proliferation, apoptosis, and immune response states (27, 38, 39). Studies have shown that stimulation with IL-15 and IL-21 can alter the miRNA profile of NK-EVs, suggesting that cargo loading is not a random process but may involve regulatory mechanisms mediated by RNA-binding proteins (40, 41). From a functional standpoint, NK-EVs cannot be simply regarded as a scaled-down version of NK cell effector functions. NK-EVs possess a more compact structure, and their biological stability is superior to that of NK cells (42, 43). Although their nanoscale size facilitates diffusion within tissues, compared with living cells, NK-EVs lack the ability to continuously sense the environment and respond in real time. Instead, their functional output primarily depends on preloaded cargo (10, 18, 27). In addition, Lugini et al. reported that NK-EVs isolated from the plasma of healthy donors exhibit cytolytic activity against cancer cells but not against resting peripheral blood mononuclear cells, and proposed that NK-EVs may have immune surveillance functions (35). Therefore, their advantages are more reflected in delivery modality, biocompatibility, and application flexibility, rather than in a mechanical amplification of function. Furthermore, NK-EVs exhibit clear source dependence and condition dependence. Differences in cellular origin, culture conditions, and isolation methods can all influence their size distribution, cargo composition, and biological effects (10, 14, 36). Collectively, the biogenesis, molecular cargo, and communication-related properties of NK-EVs are summarized in **Figure 1**. Source- and state-dependent differences relevant to function and clinical translation are compared in **Table 1**, while cross-study heterogeneity in reported NK-EVs miRNAs is analyzed in **Table 2**. Taken together, NK-EVs are therefore better considered a process- and context-sensitive class of biological products than a single entity with fixed composition or function. This heterogeneity is central to the interpretation of mechanistic studies and directly motivates the need for standardized manufacturing, characterization, and potency-assessment frameworks.

**Figure 1:**
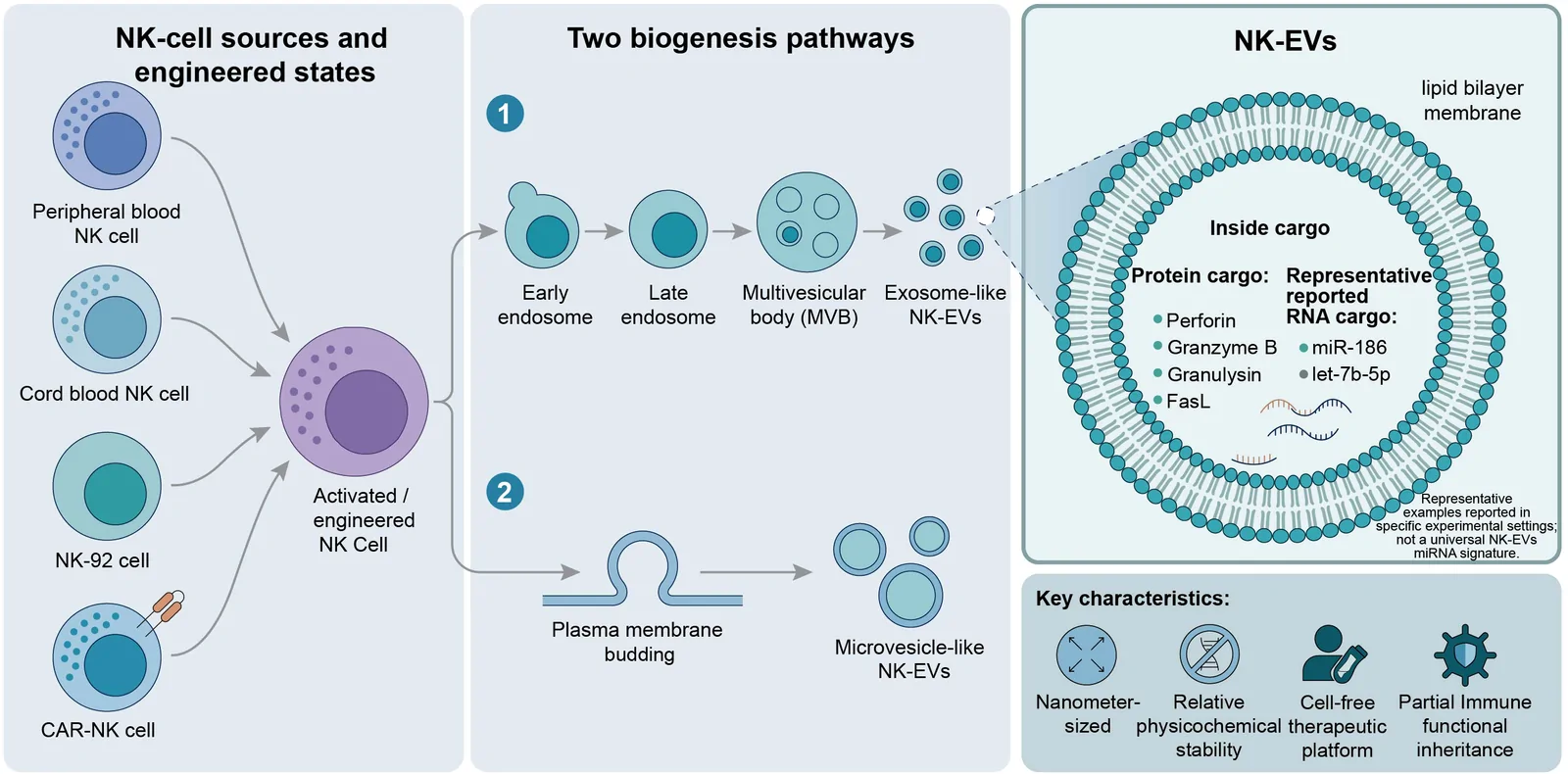
Biological characteristics, biogenesis, and communication-related cargo of NK-EVs. Biological sources commonly used for NK-EVs production include peripheral-blood NK cells, cord-blood NK cells, and NK-cell lines such as NK-92; cytokine-primed and CAR-engineered NK cells represent modified functional states rather than independent biological sources. NK-EVs preparations may contain vesicles generated through endosomal pathways and plasma-membrane budding. Representative cargos include perforin, granzymes, granulysin, FasL, and reported functional miRNAs such as miR-186 and let-7b-5p. The miRNAs shown are representative examples from specific experimental settings and should not be interpreted as a universal NK-EVs miRNA signature. Source, activation state, culture conditions, and isolation method can all alter cargo composition and signaling function.

**Table 1:** Source- and state-dependent features of NK-EVs relevant to intercellular communication.

NK-cell source/state	Main advantages	Main limitations	Manufacturing potential	Translational comment
Peripheral blood NK cells	Physiological relevance; closer to primary immune phenotype	Donor variability; limited expansion; batch heterogeneity	Moderate to low	Suitable for mechanism validation, but standardization is difficult
Cord blood NK cells	More accessible allogeneic source; expansion potential	Functional maturity and donor variability need control	Moderate	Potential source for standardized allogeneic NK-EVs
NK-92 cells	Stable cell line; high expandability; good reproducibility	Tumor-derived cell line; safety and regulatory concerns	High	Useful for preclinical production, but clinical translation requires strict safety control
Cytokine-primed NK cells	IL-15/IL-21 can enhance cytotoxic EV function and alter protein/miRNA cargo	Effects depend on stimulation scheme and culture conditions	Moderate	Useful functional-enhancement strategy, but requires potency assays
CAR-NK cells	Programmable antigen-specific recognition; potential enhancement of target-cell engagement	More complex manufacturing; antigen escape and safety evaluation remain issues	Moderate	Promising next-generation engineered NK-EVs source

Comparison of peripheral-blood NK cells, cord-blood NK cells, NK-92 cells, cytokine-primed NK cells, and CAR-engineered NK cells. The table explicitly distinguishes biological source from activation or engineered state and summarizes implications for cargo composition, reproducibility, manufacturing, recipient-cell recognition, and translational interpretation. CAR-mediated antigen recognition should not be interpreted as evidence of intrinsic tissue accumulation or BBB penetration.

**Table 2:** Cross-study comparison of miRNA profiles and functional miRNAs reported in NK-EVs.

Study	NK-cell source	Activation/stimulation	EV isolation/profiling	Representative EV-associated miRNAs	Validated target/function	Evidence level/limitation
Enomoto et al.	NK-92	None/IL-15/IL-21/IL-15+IL-21	small RNA-seq	condition-dependent profiles; miR-146b, miR-23a among responsive miRNAs	miRNA changes profiled; enhanced cytotoxicity was mechanistically linked mainly to other components such as CD226	Demonstrates stimulation-dependent heterogeneity
Dosil et al.	activated human NK cells	activated primary NK condition	small RNA sequencing	miR-10b-5p, miR-92a-3p, miR-155-5p	regulation of T-cell/Th1 responses	Functional immune-cell recipient evidence
Di Pace et al.	human NK cells	primary NK experimental system	microarray/qPCR	miR-16-5p, miR-342-3p, miR-24-3p, miR-92a-3p, let-7b-5p	let-7b-5p→CDK6; pancreatic cancer proliferation	Functional validation
Neviani et al.	NK-derived exosomes	study-specific	functional miRNA analysis	miR-186	MYCN/AURKA/TGFBR1/TGFBR2; neuroblastoma growth/immune escape	Strong molecule-specific evidence, not universal signature

Horizontal comparison of parental NK-cell source, activation or cytokine condition, EV isolation/profiling approach, representative miRNAs, validated targets or functional effects, and major limitations. The comparison highlights partial overlap across studies but supports source- and activation-state-dependent miRNA programs rather than a universal NK-EV miRNA signature.

## 3 Antitumor mechanisms mediated by NK-EVs

Based on current evidence, the antitumor effects of NK-EVs are not mediated by a single pathway, but rather arise from the integration of multiple mechanisms. From a mechanistic perspective, direct cytotoxicity mediated by perforin/granzymes has the strongest experimental basis, and Fas/FasL-mediated extrinsic apoptosis is comparatively well supported. By contrast, TRAIL–DR4/DR5 signaling remains putative in the specific context of NK-EVs. Functional miRNA transfer is supported in selected models but is source- and condition-dependent, while tumor-microenvironment modulation, anti-angiogenic effects, and anti-metastatic effects remain emerging or preliminary. **Figures 2**, **3** and **Table 3** therefore present these mechanisms using an explicit evidence hierarchy rather than as equally established pathways.

**Figure 2:**
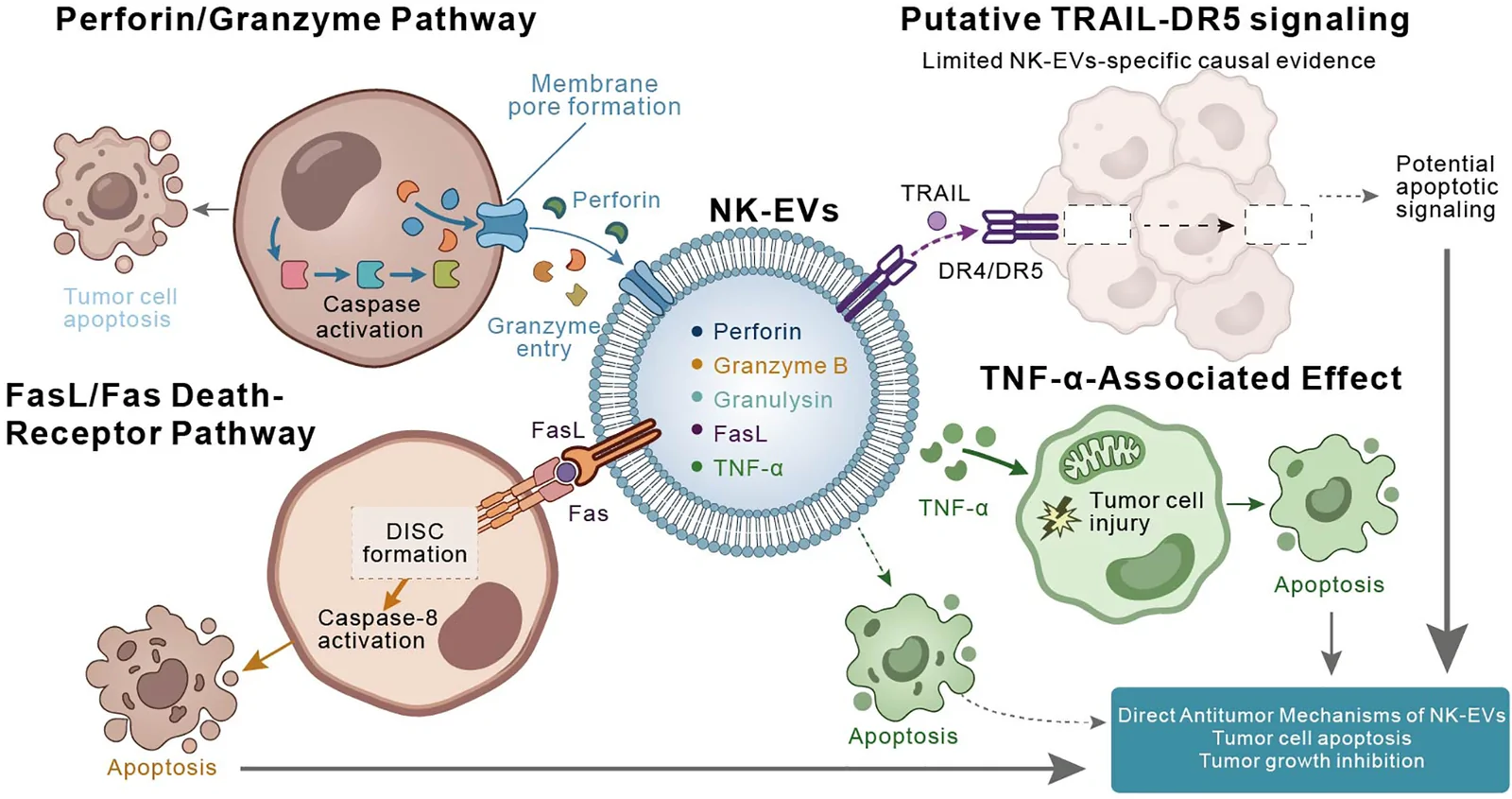
Direct antitumor mechanisms mediated by NK-EVs. NK-EVs can induce tumor-cell injury through mechanisms supported by different levels of evidence. Perforin/granzyme-mediated cytotoxicity has the strongest NK-EVs-specific support, while Fas/FasL-mediated extrinsic apoptosis is comparatively well supported. TNF-α-associated effects may contribute in selected preparations. TRAIL–DR4/DR5 is retained only as a visually de-emphasized putative pathway and should be represented by a thin dashed arrow with an explicit label indicating limited NK-EVs-specific causal evidence. Detection or theoretical presence of TRAIL is not equivalent to demonstration of DR4/DR5-dependent NK-EVs cytotoxicity. The image therefore uses visual hierarchy to distinguish established or supported mechanisms from preliminary pathways.

**Figure 3:**
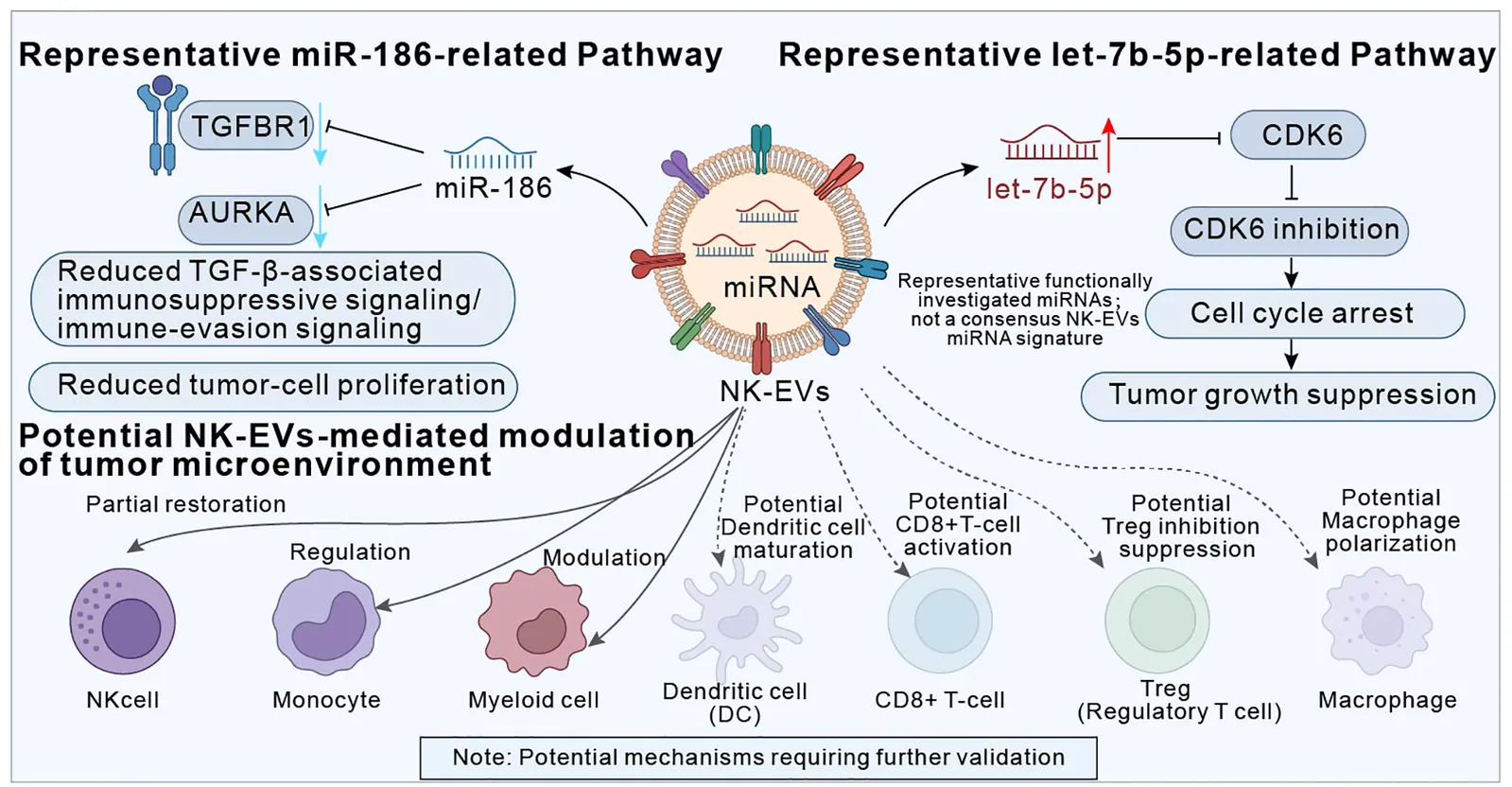
miRNA-mediated signaling regulation and potential modulation of the tumor microenvironment by NK-EVs. The miRNA pathways shown are representative functionally investigated examples rather than a consensus NK-EVs miRNA signature. miR-186 can regulate neuroblastoma-associated targets including AURKA and TGF-β pathway receptors, with effects on tumor-cell proliferation and immune-evasion signaling; these branches should be depicted separately rather than as a single linear causal chain. let-7b-5p has been linked to CDK6 inhibition and reduced pancreatic-cancer-cell proliferation. In the tumor microenvironment, NK-EVs-specific evidence supports selected effects on NK-cell activity, T cells, monocytes, and monocyte-derived dendritic cells, whereas generalized dendritic-cell maturation, CD8+ T-cell activation, Treg suppression, and macrophage polarization remain incompletely validated and should be shown with dashed arrows. Source- and state-dependent miRNA heterogeneity is compared in **Table 2**.

**Table 3:** Evidence hierarchy of signaling mechanisms currently reported for NK-EVs.

Mechanism	Key molecules/pathway	Reported biological effect	Evidence level	NK-EV-specific evidence/key limitation	Editorial interpretation
Direct cytotoxicity	Perforin, Granzyme B, Granulysin	Tumor cell membrane damage, granzyme entry, caspase activation, apoptosis	Strong	Multiple NK-EV-specific cargo and functional studies support cytotoxic-protein-mediated killing	Core and most reproducible mechanism
Death receptor signaling	FasL–Fas, DISC, Caspase-8	Extrinsic apoptosis	Moderate to strong	FasL is detected in NK-EVs and functional association is supported, although pathway-specific causal perturbation is less extensive than for granule-mediated cytotoxicity	Supported by NK-EVs studies, especially FasL-related evidence
TNF-α-associated injury	TNF-α	Tumor cell injury and death	Moderate	TNF-α has been detected/associated with cytotoxic preparations, but NK-EV-specific causal dependence is incompletely established	Should be presented as a contributing mechanism
TRAIL pathway	TRAIL–DR4/DR5	Putative extrinsic apoptosis	Preliminary/indirect	Direct evidence that EV-associated TRAIL engages DR4/DR5 and is required for NK-EV-mediated apoptosis remains insufficient	Use dashed arrows in figures; avoid overstatement
miRNA-mediated regulation	miR-186 → TGFBR1/AURKA; let-7b-5p → CDK6	Reduced proliferation, immune escape suppression, cell-cycle inhibition	Moderate, context-dependent	Selected miRNAs have functional validation, but profiles differ with NK source, activation, isolation and profiling method	Important but heterogeneous across models
Tumor microenvironment regulation	NK cell restoration; monocyte/myeloid modulation	Partial restoration of immune activity; possible immune remodeling	Preliminary to moderate	Direct support exists for selected recipient-cell effects; generalized DC/CD8/Treg/macrophage reprogramming remains incompletely validated	Promising but not yet universally validated
Anti-angiogenesis/anti-metastasis	VEGF, MMP-3, Bmi-1, PCNA downregulation	Reduced angiogenesis, migration, invasion, colony formation	Preliminary	Small number of models and mainly associative or extended phenotypes	Should be described as potential extended effects

Strong, replicated NK-EV-specific functional evidence, preferably with mechanistic perturbation or multiple models; Moderate, NK-EV-specific functional association supported in limited models; Preliminary: cargo detection, single-model observation, or incomplete causal validation; Indirect, primarily inferred from parental NK cells or other EV systems; Retracted/excluded, not used as positive supporting evidence.

Evidence-weighted summary of antitumor mechanisms attributed to NK-EVs. Perforin/granzyme-mediated cytotoxicity has the strongest NK-EV-specific support; Fas/FasL is comparatively supported; miRNA-mediated regulation is moderate but context-dependent; and TRAIL–DR4/DR5, broad tumor-microenvironment remodeling, anti-angiogenesis, and anti-metastatic effects remain preliminary or incompletely validated. Evidence level reflects NK-EV-specific functional support and causal validation rather than biological plausibility alone. Retracted findings are excluded from positive evidence synthesis.

### 3.1 Direct tumor cell killing mediated by cytotoxic molecules

Among the currently available studies on NK-EVs, the most well-supported evidence relates to their direct cytotoxic activity. Multiple studies have confirmed that NK-EVs contain a variety of cytotoxic effector molecules, including perforin, granzymes, FasL, and tumor necrosis factor-alpha (TNF-α) (16, 35). Zhu et al., in a melanoma model, demonstrated that EVs derived from NK-92 cells (originally referred to as exosomes in that study) can be internalized by tumor cells and significantly inhibit tumor growth. In this context, perforin, FasL, and TNF-α were considered to be closely associated with their cytotoxic effects (35, 44). These findings indicate, at least to some extent, that a portion of the intrinsic cytotoxic capability of NK cells can be preserved and delivered to target cells in the form of vesicles (35, 36). Mechanistically, the perforin/granzyme pathway remains central to understanding this process. Perforin increases the permeability of the target cell membrane, thereby facilitating the entry of granzymes into the cytoplasm, which subsequently activate caspase-dependent signaling cascades and ultimately induce tumor cell apoptosis (36). Previous studies have suggested that NK-EVs-induced target cell death may involve two primary mechanisms: one involves the initiation of signal transduction through the interaction between membrane-bound ligands and their corresponding receptors, and the other involves the direct fusion of vesicles with the target cell membrane, allowing the delivery of internal cargo into the cytoplasm (45–47). Although there is currently no consensus regarding the relative contribution of these two pathways, it can be generally concluded that the direct cytotoxic effect of NK-EVs is not the result of a single molecule acting in isolation, but rather a coordinated process involving multiple effector components. It should also be noted that the cytotoxic activity of NK-EVs is not fixed, but is closely related to the functional state of the parental cells. Studies have shown that small EVs derived from cytokine-induced memory-like NK cells exhibit stronger inhibitory effects on solid tumors, with increased granulysin content being considered one of the key contributing factors (48). This suggests that the cytotoxic potential of NK-EVs largely depends on the activation level and functional status of the parental NK cells. Consequently, differences in cytotoxic efficacy reported across studies are not entirely consistent. In addition, a previous study reported that NK-EVs could cross the BBB and selectively accumulate in glioblastoma tissues (RETRACTED) (49); however, this study has subsequently been retracted. Therefore, this finding should be interpreted with caution and should not be considered established evidence for the BBB crossing ability of NK-EVs. These cytotoxic processes are schematically illustrated in **Figure 2** and represent the most extensively validated antitumor mechanisms currently attributed to NK-EVs (**Table 3**). Overall, direct cytotoxic activity constitutes the most fundamental basis of NK-EVs-mediated antitumor effects and represents one of the key features that distinguish NK-EVs from EVs derived from most non-immune cells.

### 3.2 Apoptosis mediated by death receptor signaling pathways

In addition to mechanisms associated with cytotoxic granules, death receptor signaling pathways also represent an important component of NK-EVs-induced tumor cell apoptosis. Based on current evidence, the Fas/FasL axis is relatively well supported. In melanoma-related studies, FasL has been detected in exosomes derived from NK-92 cells and has been suggested to be closely associated with tumor cell death (44). According to the classical Fas/FasL signaling cascade, FasL expressed on the surface of NK-EVs may bind to Fas receptors on tumor cells, thereby inducing the formation of the death-inducing signaling complex (DISC), activating caspase-8, and initiating the extrinsic apoptotic pathway (50, 51). In contrast to the Fas/FasL pathway, direct evidence supporting the involvement of the TRAIL–DR4/DR5 pathway in NK-EVs remains relatively limited. Although TRAIL itself is an important pro-apoptotic molecule in NK cells, and there is evidence that EVs derived from other cell types can deliver TRAIL to exert antitumor effects, within the specific context of NK-EVs, current evidence remains largely at the level of potential existence rather than being fully validated (10, 27). Therefore, caution should be exercised when discussing this pathway, and conclusions drawn from other EV systems should not be directly extrapolated to NK-EVs. Taken together, NK-EVs-mediated direct cytotoxicity does not rely on a single pathway. On the one hand, NK-EVs can induce intracellular cytotoxic damage through the perforin/granzyme axis; on the other hand, membrane-associated ligands such as FasL, and potentially TRAIL, can trigger extrinsic apoptosis through receptor-dependent mechanisms. Although these pathways differ in their initial signaling events, they ultimately converge on caspase activation and cell death. This further indicates that, even in a cell-free context, NK-EVs retain certain characteristic cytotoxic functions of NK cells. A summary of perforin/granzyme-mediated cytotoxicity, Fas/FasL signaling, TNF-α-associated effects, and the potential involvement of TRAIL-related pathways is presented in **Figure 2** and **Table 3**.

### 3.3 miRNA-mediated antitumor signaling regulation

In contrast to cytotoxic proteins, which tend to exert rapid and direct killing effects, the miRNAs carried by NK-EVs are more likely to induce relatively sustained biological effects through post-transcriptional regulation within recipient cells (52, 53). These effects typically involve multiple processes, including cell proliferation, apoptosis, invasion, immune evasion, and therapeutic sensitivity (11, 38, 52, 54), and thus represent an important entry point for understanding the non-direct cytotoxic functions of NK-EVs. Among the currently reported studies, miR-186 has emerged as one of the most frequently described representative molecules. Neviani et al. demonstrated in a neuroblastoma model that miR-186 carried by NK cell-derived exosomes can enter tumor cells and downregulate multiple targets, including transforming growth factor beta receptor 1 (TGFBR1) and Aurora kinase A (AURKA) (38). In a separate primary human NK-cell study, Di Pace et al. identified a subset of miRNAs enriched in NK-cell-derived exosomes, including miR-16-5p, miR-342-3p, miR-24-3p, miR-92a-3p, and let-7b-5p, and functionally linked let-7b-5p transfer to Cyclin-dependent kinase 6 (CDK6) inhibition and reduced pancreatic-cancer-cell proliferation (55). Other studies demonstrate that NK-cell activation conditions can substantially reshape EV miRNA profiles. Enomoto et al. compared NK-92 cells cultured without cytokines or with IL-15, IL-21, or IL-15 plus IL-21 and observed cytokine-dependent changes in EV-associated miRNAs as well as proteins (41). Dosil et al. identified miR-10b-5p, miR-92a-3p, and miR-155-5p among functionally relevant miRNAs in EVs from activated primary human NK cells and linked these EVs to T-cell and myeloid-cell responses (11). These findings suggest that miRNAs carried by NK-EVs may not only influence immune-related signaling pathways but also directly participate in the regulation of tumor cell cycle progression. Representative miRNA-mediated signaling pathways currently reported for NK-EVs are summarized in **Figure 3**. However, although these studies highlight the potential value of NK-EVs in post-transcriptional regulation, the key miRNA profiles reported across different studies are not entirely consistent, suggesting that their cargo composition is still influenced by factors such as cell source, stimulation conditions, and isolation methods. Therefore, a more cautious conclusion at the current stage is that while certain miRNAs have been demonstrated to possess functional activity, it remains unclear which miRNAs are most broadly representative in NK-EVs and whether a relatively stable functional miRNA signature exists, warranting further investigation. **Table 2** provides a horizontal comparison of these studies. Thus, the most defensible current conclusion is that NK-EVs miRNA cargo reflects source- and activation-state-dependent programs, with selected miRNAs showing reproducible function in specific models rather than universal representation across NK-EVs preparations.

### 3.4 Potential modulation of the tumor immune microenvironment

The tumor microenvironment is often characterized by weakened effector immune responses, enhanced myeloid-mediated suppression, and abnormal antigen presentation, all of which collectively contribute to tumor immune evasion (54). Given that NK-EVs simultaneously carry cytotoxic proteins, membrane-associated ligands, and regulatory nucleic acids, their potential targets are unlikely to be limited to tumor cells alone, but may also include various immune cell populations (54, 56, 57). Existing evidence suggests that NK-EVs can, to some extent, influence the functional state of NK cells themselves and exert effects on monocytes or myeloid cells (58, 59). For example, in ovarian cancer-related studies, NK cell-derived EVs not only directly inhibited tumor growth but also partially restored NK cell activity under immunosuppressive conditions (60). However, current evidence regarding whether NK-EVs can consistently promote dendritic cell maturation, enhance CD8^+^ T cell activation, suppress regulatory T cell (Treg) expansion, or reprogram macrophage polarization remains limited and is largely derived from specific experimental models (11, 16, 61, 62). In other words, a more cautious conclusion at present is that NK-EVs may participate in the regulation of the tumor immune microenvironment; however, their key target cells, dominant mechanisms, and the magnitude of their *in vivo* effects remain insufficiently defined, and they are still far from being considered as a stable means of microenvironmental reprogramming. These emerging immunomodulatory effects are summarized in **Figure 3** and should be interpreted according to the evidence hierarchy presented in **Table 3**.

### 3.5 Potential anti-angiogenic and anti-metastatic effects

Compared with direct cytotoxic effects and miRNA-mediated regulation, research on the roles of NK-EVs in anti-angiogenesis and inhibition of tumor metastasis is relatively limited. Current understanding is primarily based on a small number of experimental findings and indirect inferences. Lee et al. reported that canine NK-EVs not only inhibited tumor growth but also downregulated the expression of vascular endothelial growth factor (VEGF), matrix metalloproteinase-3 (MMP-3), B-cell-specific Moloney murine leukemia virus integration site 1 (Bmi-1), proliferating cell nuclear antigen (PCNA), and various inflammation-related molecules (63). Given that angiogenesis, epithelial–mesenchymal transition, and invasion and metastasis are not independent processes, these findings suggest that the effects of NK-EVs may not be limited to inhibiting tumor cell proliferation, but may also extend to pathways associated with angiogenesis and metastatic progression (64). In addition, another study demonstrated that NK-EVs derived from cord blood can reduce cell migration, invasion, and clonogenic capacity in a dose-dependent manner in a pancreatic cancer PANC-1 model, while also exhibiting a certain degree of antitumor activity *in vivo (*65). Although these findings are noteworthy, based on the current strength of evidence, they are still insufficient to definitively classify NK-EVs as a therapeutic system with stable anti-angiogenic or anti-metastatic functions. Therefore, this aspect is more appropriately discussed as a potential extended effect of NK-EVs, rather than being assigned a high degree of certainty at the present stage. Accordingly, these activities are classified as preliminary or emerging functions in **Table 3** and are illustrated in **Figure 3**.

## 4 Functional integration of NK-EVs as intercellular signaling and cargo-transfer platforms

In addition to their intrinsic antitumor effects, NK-EVs have in recent years been increasingly recognized as functional delivery platforms with potential applications (35). Compared with conventional lipid nanoparticles or general EV-based carriers, NK-EVs are not merely inert delivery tools. Rather, their unique value lies in their dual functionality—namely, their ability to carry therapeutic cargo while retaining a certain degree of intrinsic immune effector activity (13, 36, 66, 67). From this perspective, the evaluation of NK-EVs should not focus solely on delivery efficiency, but should also assess whether their native immunological activity is preserved, and whether these two aspects can achieve functional synergy within a single system. This dual-functional concept is summarized in **Figure 4**.

**Figure 4:**
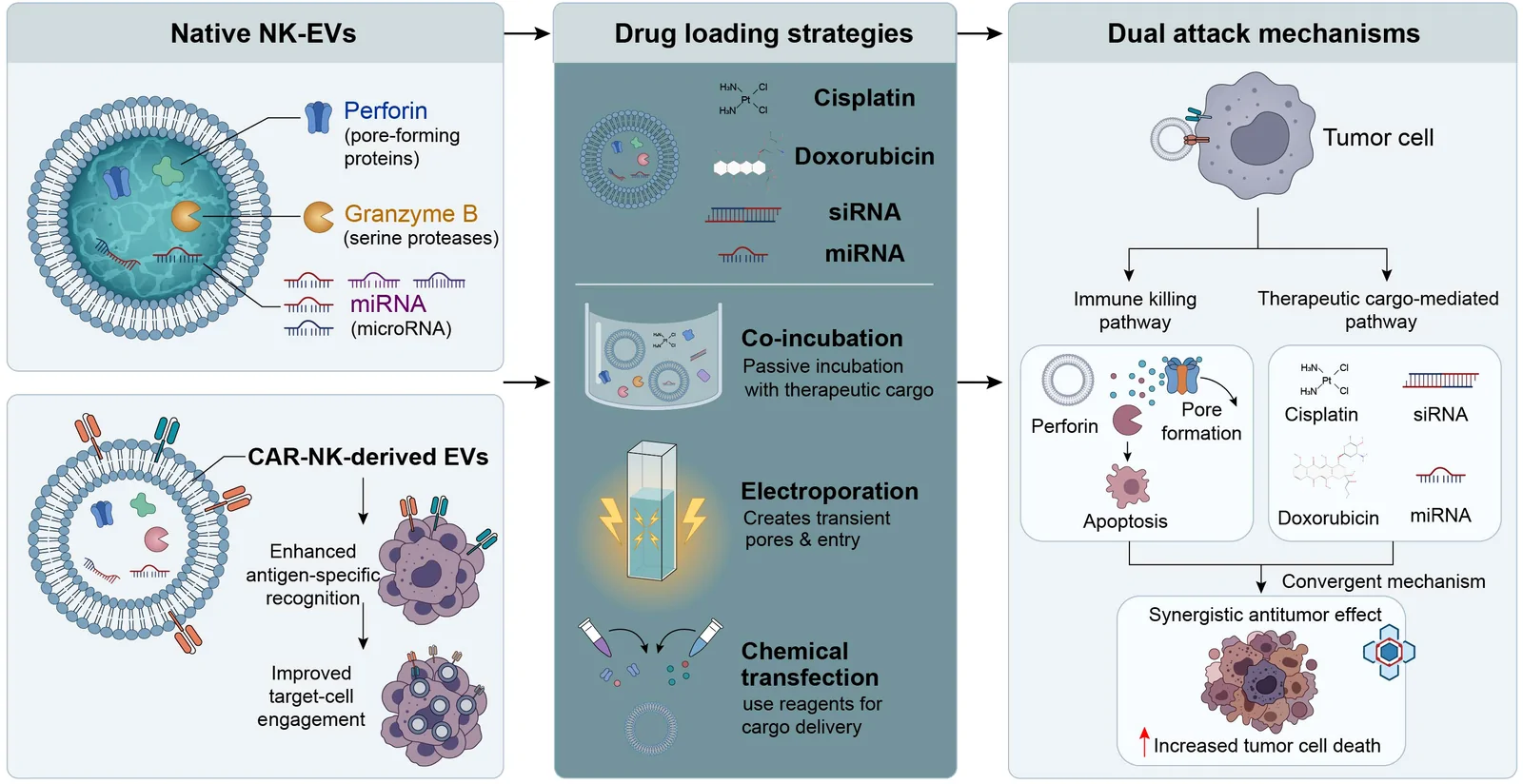
NK-EVs as intercellular signaling and cargo-transfer platforms in cancer. Native NK-EVs contain endogenous immune-effector and regulatory cargos and can be combined with exogenous therapeutic cargo. CAR-bearing NK-EVs may enhance antigen-specific target-cell recognition, but CAR expression should not be equated with improved tissue biodistribution or BBB penetration. In the reported HER2-positive brain-metastasis platform, BBB-associated delivery was achieved in a composite ExoCAR/T7@Micelle system in which T7 was introduced for transferrin-receptor-mediated BBB targeting, whereas CAR primarily mediated HER2-specific recognition (68). The image should therefore separate antigen-recognition engineering from tissue/barrier-targeting engineering and avoid depicting CAR-NK-derived EVs alone as intrinsically brain-targeting or as necessarily showing improved tumor accumulation.

Drug loading represents one of the primary approaches to achieving such functional integration. Taking chemotherapeutic agents as an example, studies in ovarian cancer have shown that cisplatin-loaded NK-EVs not only enhance tumor cell sensitivity to the drug but also, to some extent, restore suppressed NK cell function (13, 60, 68). In hepatocellular carcinoma models, doxorubicin-loaded NK-EVs exhibit stronger cytotoxic and pro-apoptotic effects compared with free drug or unloaded vesicles, while still retaining effector molecules such as granzyme B and perforin (69). These findings indicate that the advantage of NK-EVs lies not merely in delivering therapeutic agents into tumor cells, but also in their ability to integrate the effects of exogenous drugs with the intrinsic immune-mediated cytotoxicity of the vesicles themselves within a single delivery unit, thereby generating synergistic antitumor effects. Representative therapeutic delivery strategies based on NK-EVs are illustrated in **Figure 4**.

On this basis, parental cell engineering further expands the functional scope of NK-EVs. A key example is the brain-metastasis study by Tao et al, but its mechanistic interpretation requires an important distinction (70). CAR-NK-derived exosomes were incorporated into an ExoCAR/T7@Micelle platform, in which T7 facilitated BBB transport, whereas CAR expression primarily mediated HER2-specific tumor-cell recognition. Thus, the observed BBB delivery should not be interpreted as evidence of an intrinsic BBB-crossing capacity of CAR-NK-derived EVs. More broadly, antigen recognition, recipient-cell uptake, tissue biodistribution, and barrier penetration are distinct properties and should be evaluated independently. Most reported delivery advantages remain preclinical and must be validated with quantitative biodistribution, pharmacokinetic, and safety studies before translational conclusions can be drawn (69, 70). In particular, both drug loading and surface modification processes may affect vesicle membrane integrity and the composition of surface proteins (66). If the intrinsic immune activity is consequently compromised, improvements in delivery performance may not necessarily translate into overall therapeutic benefit. Therefore, improved delivery or target-cell engagement should be interpreted together with preservation of intrinsic immune activity and product identity (**Figure 4**; **Tables 4**, **5**).

**Table 4:** Comparison of NK-EVs engineering strategies.

Strategy	Representative approaches	Intended purpose	Advantages	Key risks/limitations	Translational maturity
Parent-cell priming	IL-15, IL-21 stimulation	Enhance endogenous cytotoxic cargo and functional activity	Less direct disruption of EVs membrane; preserves natural features	Cytokine response depends on NK source and culture system	Preclinical — intermediate
Cargo loading	Cisplatin, doxorubicin, siRNA, miRNA	Combine immune killing with drug-induced cytotoxicity	Enables synergistic therapy	Loading may alter membrane integrity, uptake, biodistribution and pharmacokinetics	Preclinical — intermediate
Antigen-recognition engineering	CARs, antibodies, antigen-binding ligands	Increase antigen-specific target-cell recognition or engagement	Programmable recipient-cell specificity	Antigen escape; altered membrane organization; recognition does not establish enhanced tissue accumulation	Preclinical — early/intermediate
Tissue/barrier-targeting engineering	T7 or other tissue/barrier-targeting ligands in engineered composite systems	Modify tissue delivery or physiological-barrier transport	Can add tissue-specific delivery mechanisms distinct from antigen recognition	Must demonstrate quantitative biodistribution and barrier transport; engineered delivery must not be generalized to native NK-EVs	Preclinical—early
Biomimetic NK-EVs	Cell extrusion, artificial NK-derived vesicles	Improve yield, scalability and batch production	Potentially higher production efficiency	Biological equivalence to natural NK-EVs remains uncertain	Preclinical — early
Formulation/stabilization	Hydrogel, microneedle or protective biomaterial systems	Improve stability and delivery feasibility	May improve storage and local delivery	Requires additional safety and release-control validation	Preclinical — early

Antigen recognition, target-cell uptake, tissue accumulation and BBB penetration are treated as distinct properties. All maturity labels refer to preclinical development.

Comparison of principal NK-EV engineering strategies, with antigen-recognition engineering separated from tissue/barrier-targeting engineering and NK-cell-derived artificial nanovesicles distinguished from naturally secreted NK-EVs. Translational maturity is described explicitly as preclinical. The table emphasizes that improvements in recognition, biodistribution, cargo delivery, scalability, or stability require parallel assessment of product identity, intrinsic activity, pharmacokinetics, and comparability.

**Table 5:** Comparative translational and regulatory considerations for NK-EVs, other EV-based therapeutics, and CAR-T/CAR-NK products.

Dimension	Native/engineered NK-EVs	Other therapeutic EVs	CAR-T/CAR-NK	Implication for NK-EV development
Product identity	Complex acellular vesicle product	Source-/cargo-dependent	Living genetically modified cells	Define active substance and MoA early
Regulatory positioning	Product-specific; no dedicated harmonized NK-EV pathway	Drug/biologic or other category depending jurisdiction/product	Gene/cell therapy framework more mature	Early regulator consultation required
Starting material	NK source + culture/activation state	Donor/cell-source dependent	T/NK cells + vector/editing system	Source-cell qualification critical
Potency	Not standardized; MoA-linked assay needed	Product-specific	Established expectation for product-specific potency	Combine cytotoxicity/targeting/cargo-function assays
Release testing	EV identity, purity, contamination, cargo/activity	Similar EV CQA issues	Identity, purity, viability, potency, sterility etc.	Build NK-EV-specific CQA matrix
Biodistribution	Major unresolved issue	Also important	Cellular trafficking/persistence	PK/biodistribution must be characterized
Genetic engineering	May alter EV cargo/surface/transgene-associated risk	Depends on source engineering	Central regulatory feature	CAR-NK-EVs may require additional gene-therapy-related considerations
Manufacturing comparability	Major challenge	Major challenge	Relatively mature comparability concepts	Apply lifecycle comparability principles

Comparison of product definition, regulatory positioning, starting-material control, CQAs and release testing, potency, biodistribution, genetic engineering, manufacturing comparability, and safety. The table emphasizes that NK-EV regulation is product- and jurisdiction-specific rather than absent and uses more developed EV and cell/gene-therapy frameworks as translational comparators without implying direct regulatory equivalence.

## 5 Engineering strategies for NK-EVs

Engineering strategies for NK-EVs should be interpreted not only as approaches to enhance delivery efficiency, but also as methods to modulate vesicle-mediated signaling capacity, cargo composition, cellular recognition, and tumor–immune communication (71, 72). With the continuous advancement of research, reliance solely on native NK-EVs has become insufficient to meet the demands for functional enhancement and industrial-scale application. Current engineering strategies, aimed at improving antitumor activity, delivery efficiency, and large-scale manufacturability, mainly include parental cell priming, exogenous cargo loading, surface targeting modification, and biomimetic construction. These strategies differ in their primary focus: some aim to enhance intrinsic biological effects, others emphasize functional integration, while some attempt to address targeting limitations and manufacturing bottlenecks. The major engineering strategies currently applied to NK-EVs are summarized in **Figure 5** and compared in **Table 4**.

**Figure 5:**
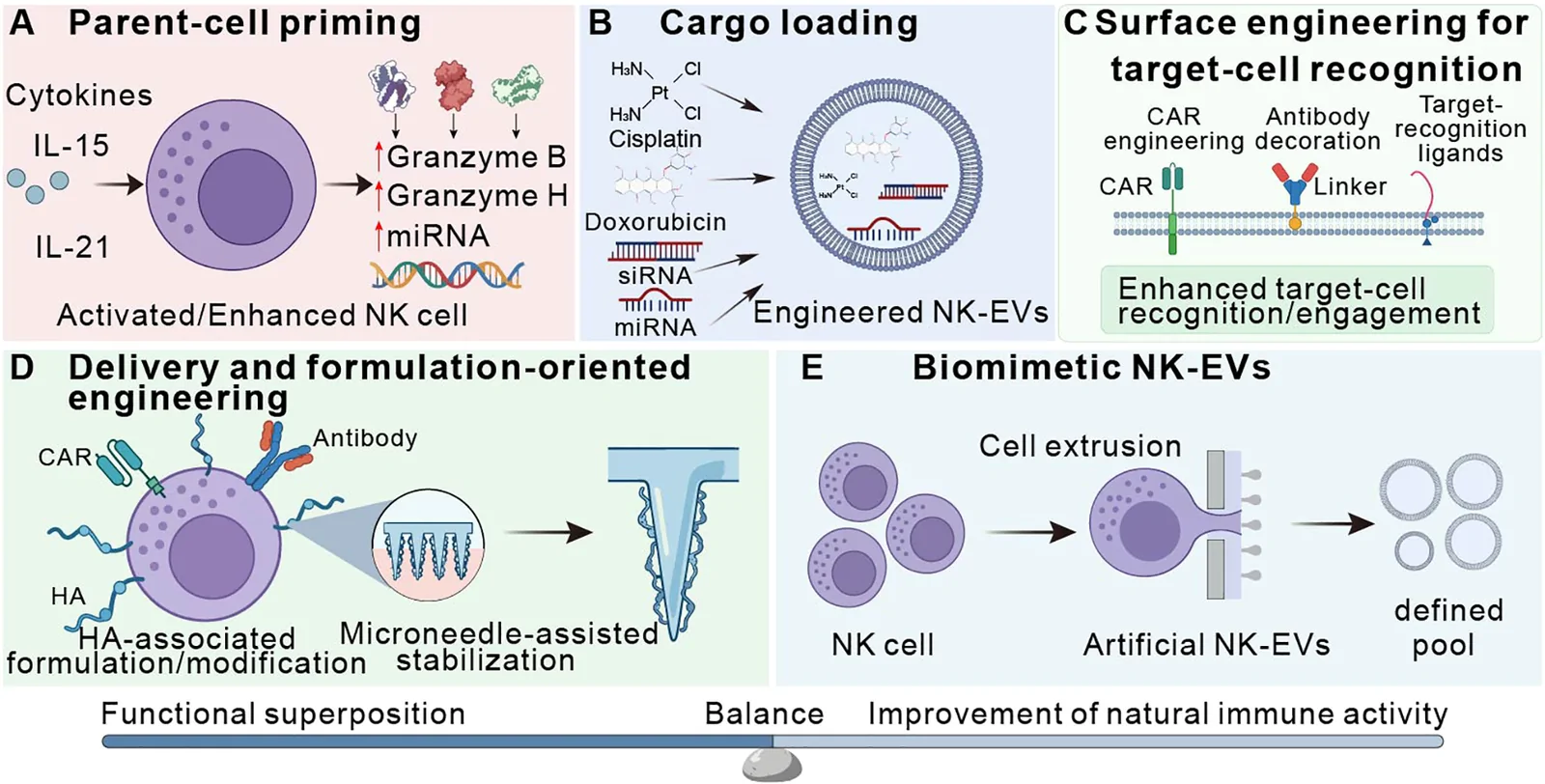
Engineering strategies for modulating NK-EVs signaling capacity and functional communication. Engineering approaches should be presented as five mechanistically distinct categories: **(A)** parental-cell priming, which changes the functional state of the NK cell and thereby alters EV cargo; **(B)** exogenous cargo loading; **(C)** surface/recognition and tissue-targeting engineering, with antigen recognition distinguished from barrier/tissue delivery; **(D)** NK-cell-derived artificial or biomimetic nanovesicle construction, which should be labeled separately from naturally secreted NK-EVs; and **(E)** formulation and stabilization strategies such as protective excipients or microneedle-assisted local delivery. The central design principle is a balance between engineered functional gain and preservation of native or intended product identity, cargo integrity, and mechanism-linked activity.

### 5.1 Parental cell priming

Modulating the state of parental cells to indirectly alter vesicle properties represents an engineering approach with relatively low intervention intensity and is widely adopted. This strategy typically employs cytokine stimulation to regulate the activation level of NK cells, thereby modifying the protein and miRNA composition of secreted vesicles without directly disrupting vesicle structure. Existing studies have demonstrated that cytokines such as IL-15 and IL-21 can significantly enhance NK cell proliferation and cytotoxic activity, and further influence the functional characteristics of their secreted vesicles (22, 73, 74). Enomoto et al. reported that combined stimulation with IL-15 and IL-21 upregulated the expression of granzyme B, granzyme H, and related miRNAs in NK-92-derived exosomes, and significantly enhanced their ability to induce tumor cell apoptosis (41). Similar observations have been reported in hepatocellular carcinoma models, where cytokine pretreatment improved the antitumor effects of NK-EVs (75). The advantage of this strategy lies in its minimal direct interference with vesicle membrane structure and surface molecules, thereby facilitating the preservation of intrinsic immune characteristics. However, its limitations are also evident: the extent of functional enhancement is highly dependent on parental cell source, stimulation protocols, and culture systems, resulting in limited reproducibility and standardization (76). At present, parental cell priming is more appropriately regarded as an adaptive optimization approach rather than a precisely controllable engineering strategy. Parent-cell priming is illustrated in **Figure 5** and compared with other engineering approaches in **Table 4**.

### 5.2 Exogenous cargo loading

Exogenous cargo loading represents the most direct strategy for enhancing the functionality of NK-EVs. Through methods such as co-incubation, electroporation, or chemical transfection, small-molecule drugs, siRNAs, or miRNAs can be introduced into vesicles, thereby constructing composite therapeutic systems that integrate endogenous immune cytotoxicity with exogenous therapeutic interventions (59, 62, 77, 78). Studies have shown that doxorubicin-loaded EVs exhibit superior antitumor effects compared with free drugs across multiple tumor models (77, 79). In the specific context of NK-EVs, similar strategies not only improve drug delivery efficiency but also retain key effector molecules such as granzyme B and perforin, thereby enabling synergistic effects between chemotherapy and immune-mediated cytotoxicity (59, 80). From a mechanistic perspective, this strategy integrates the perforin/granzyme pathway mediated by NK cells with drug-induced cellular damage, thereby offering the potential for multi-pathway synergistic tumor targeting (53). However, it should be noted that the loading process may alter vesicle membrane integrity and surface protein composition, which in turn may affect cellular uptake mechanisms, *in vivo* distribution, and pharmacokinetic behavior (54, 59). In addition, the stability of drug delivery carriers is a critical factor influencing the overall efficacy of delivery systems (81). Therefore, evaluation of cargo-loaded NK-EVs should not be limited to loading efficiency alone, but should also systematically assess the preservation of intrinsic immune activity, changes in surface characteristics, and potential alterations in *in vivo* behavior. The advantages and limitations of cargo loading strategies are summarized in **Figure 5** and **Table 4**.

### 5.3 Surface targeting modification

Surface modification is primarily employed to enhance the ability of NK-EVs to recognize target tissues and improve delivery efficiency *in vivo (*82, 83). Although NK-EVs possess inherent advantages derived from their immune origin, their *in vivo* distribution often lacks sufficient specificity, resulting in limited tumor accumulation. To address this limitation, some studies have utilized genetic engineering approaches to modify parental cells, enabling the vesicles they secrete to express specific targeting molecules (13, 82). For example, EVs derived from CAR-engineered immune cells have been developed to enhance recognition of specific tumor antigens (70, 84, 85). In neuroblastoma models, Neviani et al. demonstrated that miR-186 carried by NK-EVs exerts antitumor effects. Additionally, Haichang Shun et al. employed CD56 antibody-coated nanoparticles to deliver regulatory molecules to NK cells, and found that this approach could partially alleviate TGF-β-mediated functional suppression. These findings suggest that miR-186 may not only function as an endogenous component of NK-EVs but may also participate in the modulation of NK cell-based therapies (38). Furthermore, modifications involving biomaterials such as hyaluronic acid and trehalose-based dissolvable microneedles may help improve the stability of NK-EVs (86). It is important to recognize that membrane proteins play essential roles in cellular recognition, uptake, and immune regulation. Excessive or inappropriate modification may interfere with the natural biological behavior of vesicles (87, 88). Therefore, the key challenge of surface targeting modification lies not in the ability to introduce targeting molecules, but in achieving enhanced targeting while minimizing disruption to the intrinsic functional characteristics of NK-EVs. Surface-targeting modifications are schematically illustrated in **Figure 5**.

### 5.4 Biomimetic construction

Biomimetic construction has emerged as another area of increasing interest in recent years. Native NK-EVs suffer from limitations such as low yield and significant batch variability, which directly affect their prospects for clinical translation (80). To address manufacturing bottlenecks, researchers have attempted to construct artificial NK-like vesicles using methods such as cell extrusion, thereby improving production efficiency and scalability (80, 89, 90). Studies have reported that vesicle-like structures generated through cell extrusion can retain certain membrane proteins, and subsequent investigations have indicated that these biomimetic vesicles exhibit advantages in terms of *in vitro* antitumor activity and scalability, while also being capable of enriching functional proteins such as granzyme B (89, 91, 92). More recent studies have shown that biomimetic NK-EVs can recruit immune cells and suppress oncogene expression, thereby exerting synergistic antitumor effects (46, 93, 94). However, although biomimetic strategies provide new approaches from a manufacturing perspective, their biological equivalence remains a critical issue. It is still unclear whether artificially constructed vesicles can faithfully replicate native NK-EVs in terms of membrane integrity, cargo loading selectivity, and *in vivo* behavior (13, 95). In particular, whether artificial vesicles can reproduce the complex immunoregulatory functions of natural NK-EVs remains far from being conclusively determined. Overall, although the above engineering strategies each have distinct focuses, they also face a common challenge: any enhancement of functionality may be accompanied by a loss of intrinsic biological properties. Future efforts should therefore place greater emphasis not only on continuously adding new functions, but also on achieving a balance between controllable engineering modification and the preservation of native immune activity. A comparison of current engineering approaches and their translational maturity is provided in **Table 4**.

## 6 Biological and translational challenges in defining NK-EVs-mediated communication

The factors limiting the clinical translation of NK-EVs do not stem from a single isolated issue, but rather from the lack of an integrated technical framework encompassing multiple stages, including isolation and purification, large-scale production, *in vivo* delivery, and safety evaluation. In other words, what is lacking for NK-EVs to become true clinical products is not merely a breakthrough in a single aspect, but the establishment of a complete, mature, reproducible, and regulatory-compliant development pipeline. The major translational barriers currently facing NK-EVs are summarized in **Figure 6**.

**Figure 6:**
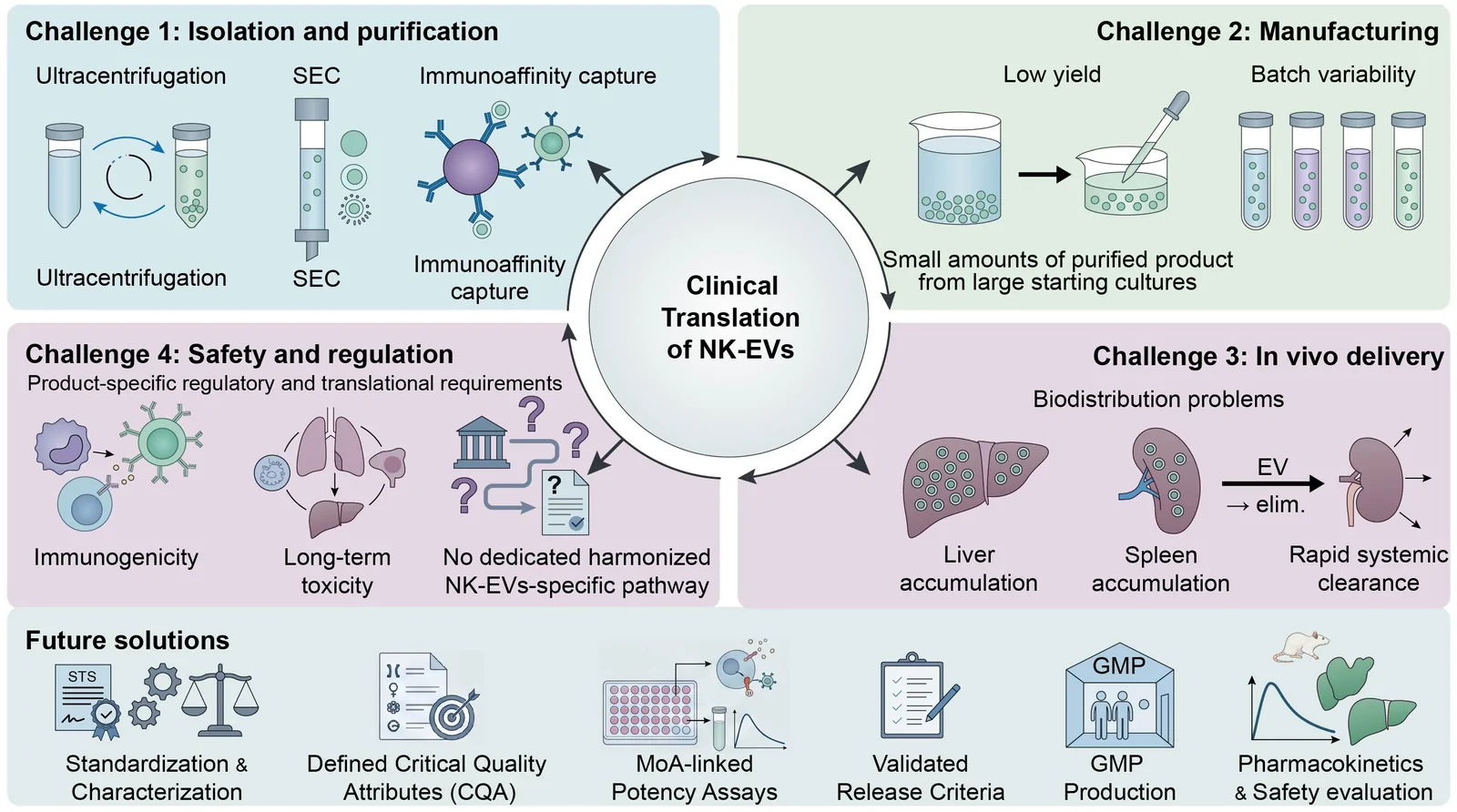
Biological and translational challenges in defining NK-EVs-mediated tumor–immune communication. Several interconnected challenges limit NK-EVs translation: (1) isolation and purification variability; (2) source-, activation-, and process-dependent manufacturing heterogeneity; (3) uncertain biodistribution, pharmacokinetics, and clearance; and (4) incomplete repeated-dose safety data together with product-specific regulatory classification and the absence of a harmonized NK-EVs-specific release framework. The regulatory panel should not imply a regulatory vacuum. Future development requires defined CQAs, mechanism-linked potency assays, validated lot-release criteria, reproducible GMP-compatible manufacturing, quantitative biodistribution studies, toxicology, comparability strategies, and early alignment with the relevant regulatory framework.

### 6.1 Lack of standardization in isolation, purification, and quality control systems

NK cell culture supernatants contain a large number of soluble proteins and non-vesicular particles, which makes the preparation of high-purity NK-EVs inherently technically challenging (13, 95). Currently used methods, including ultracentrifugation, size-exclusion chromatography, and immunoaffinity capture, involve trade-offs among purity, recovery rate, and vesicle integrity (96). More importantly, different isolation strategies may alter the composition and functional characteristics of the resulting EV subpopulations, thereby reducing comparability across studies (12, 48). Although MISEV2018 and its updated version MISEV2023 have further emphasized the need for standardized nomenclature, isolation, characterization, and functional evaluation of EVs, no unified consensus has yet been established in the field of NK-EVs regarding source control, processing workflows, potency assays, and the definition of critical quality attributes (22, 33, 97). Therefore, establishing robust isolation and purification protocols, along with reproducible quality control systems, is not only a prerequisite for standardized research but also a fundamental requirement for subsequent clinical translation. Challenges associated with isolation, purification, and quality control are highlighted in **Figure 6**.

### 6.2 Limitations in large-scale production and batch-to-batch consistency

The yield of NK-EVs is influenced by multiple factors, including cell source, expansion efficiency, and activation status (98, 99). Moreover, the quantity required for clinical applications is far greater than that typically used in laboratory-scale research (100). In practice, obtaining sufficient numbers of functionally stable NK cells from blood sources is itself challenging (101, 102). Although cytokine preactivation and bioreactor-based culture systems can improve production yield, they may introduce additional sources of variability, thereby affecting batch-to-batch consistency (97, 103, 104). For NK-EVs, the primary challenge lies not only in insufficient yield, but also in maintaining relative stability in size distribution, cargo composition, biological activity, and safety during scale-up production (12, 97). If these parameters cannot be consistently controlled, even high yields would be insufficient to support downstream pharmaceutical development and clinical evaluation. Therefore, scalability and consistency should not be viewed as separate issues, but rather as two interrelated aspects of clinical manufacturability. Manufacturing scalability and batch consistency represent key components of the translational framework summarized in **Figure 6**.

### 6.3 Insufficient *in vivo* distribution and delivery efficiency

Existing studies indicate that EVs administered intravenously are readily cleared by the mononuclear phagocyte system and predominantly accumulate in organs associated with the reticuloendothelial system, such as the liver and spleen. This phenomenon may be attributed to factors including phagocytic recognition of vesicle surfaces, adsorption of plasma proteins, and limited circulation stability, ultimately resulting in insufficient exposure within tumor tissues (105). Although some studies suggest that NK-EVs may possess certain tumor-targeting tendencies or advantages derived from their immune origin, it remains unclear whether these characteristics are stable, broadly applicable, or significantly influenced by different routes of administration. Therefore, improving the delivery efficiency of NK-EVs to tumor sites and clarifying their *in vivo* distribution, clearance pathways, and pharmacokinetic profiles are essential prerequisites for clinical application. Without such foundational data, the design of dosing regimens, evaluation of therapeutic efficacy, and assessment of safety risks cannot be reliably established. These issues are included among the delivery-related barriers summarized in **Figure 6**.

### 6.4 Safety, product definition, and regulatory considerations

Current safety evidence for NK-EVs is derived mainly from *in vitro* studies and animal models, with limited information on repeated dosing, long-term toxicity, immunogenicity, thrombogenicity, off-target biodistribution, or consequences of process-related impurities. Residual source-cell material, culture-medium components, non-vesicular particles, adventitious agents, and engineered cargos may alter the safety profile of the final preparation (106–108). These risks argue for product-specific specifications and validated assays rather than a generic assumption that a cell-free product is intrinsically safe.

NK-EVs are not in a regulatory vacuum, but there is no single harmonized NK-EVs-specific regulatory pathway that applies across all product configurations and jurisdictions. In the United States, the FDA has stated that exosome products intended to treat disease are, as a general matter, regulated as drugs and biological products and are subject to premarket review; the agency also states that there are currently no FDA-approved exosome products (109). ISEV has emphasized that EV-based therapeutics should be categorized within existing regulatory frameworks according to whether EVs constitute the active substance or primarily serve as a delivery vehicle, and that manufacturing and quality-control requirements follow from that product definition (107). The joint ISCT/ISEV statement likewise stresses the need for appropriate manufacturing and quality control, preclinical safety and efficacy data, rational clinical-trial design, and regulatory oversight before therapeutic EV use (108). In Europe, EMA classification examples further illustrate the product-specific nature of regulation: exosomes carrying recombinant mRNA and miRNA have been classified as a gene therapy medicinal product, whereas expanded allogeneic NK cells have been classified as a somatic cell therapy medicinal product (110). Thus, regulatory classification depends on composition, active substance, engineering strategy, mechanism of action, and jurisdiction rather than on the label ‘EV’ alone.

CAR-T/CAR-NK development provides a useful comparator but should not be presented as directly interchangeable with NK-EV regulation. FDA’s 2024 CAR-T guidance provides detailed recommendations for CMC, pharmacology/toxicology, clinical development, and analytical comparability and notes that many principles are also applicable to other genetically modified lymphocyte products, including CAR-NK cells (111). Potency expectations are likewise product-specific: FDA guidance states that potency measurements are designed for a particular product and does not prescribe a universal assay or release criterion (112). For NK-EVs, a mechanism-of-action-linked potency strategy is therefore more defensible than a single universal marker. Native cytotoxic NK-EVs may require functional tumor-cell-killing assays linked to cytotoxic cargo; CAR-bearing EVs may additionally require antigen-binding or target-cell-recognition assays; and drug-loaded NK-EVs may require cargo content/release measurements together with preserved EV identity and intrinsic activity. Across these configurations, release testing should address identity, purity, particle attributes, process impurities, microbiological safety, cargo consistency, functional potency, stability, and lot comparability. **Table 5** summarizes these translational and regulatory parallels and differences.

## 7 Conclusion and perspectives

The antitumor effects of NK-EVs are not limited to a single mechanism. Based on current evidence, perforin/granzyme-mediated cytotoxicity currently has the strongest NK-EVs-specific support, and Fas/FasL signaling is comparatively well supported. TRAIL–DR4/DR5 signaling should remain classified as putative until direct pathway-specific causal evidence is available.

Functionally characterized miRNAs such as miR-186 and let-7b-5p demonstrate the capacity of NK-EVs to mediate post-transcriptional regulation, but cross-study comparison indicates source- and activation-state-dependent miRNA programs rather than a stable universal NK-EV signature. NK-EVs are also being developed as therapeutic cargo carriers; however, engineered improvements in antigen recognition or tissue-specific delivery should be attributed to the specific engineering element and should not be generalized as intrinsic properties of native NK-EVs. In particular, current evidence supports engineered BBB-targeting composite systems rather than established intrinsic BBB crossing by native or CAR-NK-derived EVs.

At present, the primary factors limiting further development of NK-EVs are still unresolved fundamental issues. It remains unclear whether NK-EVs derived from different NK cell sources, culture systems, and isolation methods can achieve relatively stable and reproducible differences in composition and function. Without resolving this issue, subsequent functional evaluations and cross-study comparisons may be significantly affected. At the same time, large-scale production, quality control, and batch consistency remain unavoidable challenges, as the development of any stable therapeutic product must be based on reproducible manufacturing processes. Furthermore, aspects more closely related to clinical translation—such as *in vivo* distribution, metabolic fate, tissue exposure levels, and long-term safety—are still supported by insufficient evidence. Therefore, future research priorities may not lie in continuously expanding application scenarios, but rather in strengthening several key foundational aspects. First, standardization of isolation, characterization, and quality evaluation methods would improve comparability across studies and facilitate process scale-up and regulatory development. Second, there is still a lack of sufficiently clear data regarding the *in vivo* distribution, clearance, and duration of action of NK-EVs, which directly affects dosing strategy design and efficacy assessment. Third, although engineering modifications can enhance functionality, it is equally important to evaluate whether such modifications compromise intrinsic immune activity. Rather than focusing solely on maximizing targeting efficiency or loading capacity, greater emphasis should be placed on achieving a balance between controllable engineering modification and preservation of native biological function. The key translational bottlenecks and future priorities are summarized in **Figure 6** and **Tables 1**, **3**, **4**.

Overall, NK-EVs represent a specialized mode of immune-cell-derived intercellular communication in cancer, but their therapeutic interpretation requires explicit separation of intrinsic vesicle biology from properties conferred by parental-cell engineering, post-isolation modification, artificial nanovesicle construction, or formulation. An evidence-weighted framework that links NK-cell source and state to EV cargo, recipient-cell specificity, signaling mechanism, biodistribution, potency, and product definition provides a more rigorous basis for future development. Progress will depend less on expanding mechanistic claims than on validating causal pathways, defining reproducible source- and process-dependent product attributes, and establishing quantitative *in vivo* and regulatory-compliant evaluation systems.

## Glossary

ADC: Antibody–drug conjugate
AURKA: Aurora kinase A
BBB: Blood–brain barrier
CAR: Chimeric antigen receptor
CAR-NK: Chimeric antigen receptor-engineered natural killer cell
CMC: Chemistry, manufacturing, and controls
CQA: Critical quality attribute
CDK6: Cyclin-dependent kinase 6
DC: Dendritic cell
DISC: Death-inducing signaling complex
DR4: Death receptor 4
DR5: Death receptor 5
EMA: European Medicines Agency
EVs: Extracellular vesicles
FDA: U.S. Food and Drug Administration
FasL: Fas ligand
GMP: Good manufacturing practice
IL: Interleukin
MVB: Multivesicular body
miRNA: MicroRNA
MoA: Mechanism of action
NK cell: Natural killer cell
NK-EVs: Natural killer cell-derived extracellular vesicles
NK-92: NK-92 cell line
PBMC: Peripheral blood mononuclear cell
PB-NK: Peripheral blood-derived natural killer cell
RNA: Ribonucleic acid
SEC: Size-exclusion chromatography
siRNA: Small interfering RNA
TGFBR1: Transforming growth factor beta receptor 1
TfR: Transferrin receptor
TME: Tumor microenvironment
TNF-α: Tumor necrosis factor-alpha
TRAIL: TNF-related apoptosis-inducing ligand
Treg: Regulatory T cell.
